# Stored alcohol and fatty acid intermediates and the biosynthesis of sex pheromone aldehyde in the moth *Chloridea virescens*

**DOI:** 10.1007/s10886-024-01478-x

**Published:** 2024-02-20

**Authors:** Stephen P. Foster, Karin G. Anderson

**Affiliations:** Department of Entomology, School of Natural Resource Sciences, North Dakota State University, 7650, PO Box 6050, Fargo, ND 58108-6050 USA

**Keywords:** Lepidoptera, Tracer-tracee, Stable isotopes, PBAN, Pheromone dynamics, Fat synthesis

## Abstract

In most species of moths, the female produces and releases a volatile sex pheromone from a specific gland to attract a mate. Biosynthesis of the most common type of moth sex pheromone component (Type 1) involves de novo synthesis of hexadecanoate (16:Acyl), followed by modification to various fatty acyl intermediates, then reduction to a primary alcohol, which may be acetylated or oxidized to produce an acetate ester or aldehyde, respectively. Our previous work on the moth *Chloridea virescens* (Noctuidae) showed that females produce 90% of the major pheromone component, (*Z*)-11-hexadecenal (Z11-16:Ald), via a direct and rapid route of de novo biosynthesis with highly labile intermediates, and ca. 10% from an indirect route that likely mobilizes a pre-synthesized 16-carbon skeleton, possibly, (*Z*)-11-hexadecenoate (Z11-16:Acyl) or hexadecanoate (16:Acyl). In this paper, we use stable isotope tracer/tracee techniques to study the dynamics of the precursor alcohol (*Z*)-11-hexadecenol (Z11-16:OH) and stores of Z11-16:Acyl and 16:Acyl to determine their roles in biosynthesis of Z11-16:Ald. We found: (i) that intracellular Z11-16:OH is synthesized at roughly the same rate as Z11-16:Ald, indicating that translocation and oxidation of this moiety does not rate limit biosynthesis of Z11-16:Ald, (ii) intracellular Z11-16:OH consists of two pools, a highly labile one rapidly translocated out of the cell and converted to Z11-16:Ald, and a less labile one that mostly remains in gland cells, (iii) during pheromone biosynthesis, net stores of Z11-16:Acyl increase, suggesting it is not the source of Z11-16:Ald produced by the indirect route, and (iv) no evidence for the gland synthesizing stored 16:Acyl prior to (up to 2 days before eclosion), or after, synthesis of pheromone commenced, suggesting the bulk of this stored moiety is synthesized elsewhere and transported to the gland prior to gland maturation. Thus, the pheromone gland of *C*. *virescens* produces very little stored fat over its functional lifetime, being optimized to produce sex pheromone.

## Introduction

Mating in moths is characterized by release of a volatile sex pheromone that facilitates recognition and location of mates over distance (Allison and Cardé 2016). In most species, the female biosynthesizes and releases sex pheromone from a specialized gland, typically located on an intersegmental membrane between the 8th and 9th abdominal segments (Ma and Ramaswamy 2003). The most common type of moth sex pheromone component is a 10–16, even numbered-carbon chain with terminal oxygenated functionality, and one or more positions of unsaturation, known as “Type 1” (Ando and Yamamoto 2020). These compounds are formed by de novo biosynthesis of hexadecanoate (16:Acyl) from acetyl CoA, followed by specific reactions that shape chain length, unsaturation and functionality (Blomquist et al. 2011; Foster 2016; Jurenka 2020). In many species, biosynthesis is controlled by circadian release of the pheromone biosynthesis-activating neuropeptide (PBAN) which, depending on species, may control the synthesis of 16:Acyl or the reduction of fatty acyl intermediates (Jurenka 2020; Matsumoto et al. 2002; Rafaeli 2009).

To release pheromone, female moths adopt a characteristic posture, known as calling, in which the ovipositor is extruded, and the gland everted, allowing pheromone on the surface to move into the airstream (McNeil 1991). When females do not call, but are biosynthesizing pheromone, the gland stores pheromone, enabling it to release at a higher rate during the next calling bout (Foster and Anderson 2020b, 2022). In addition to pheromone, moth sex pheromone glands often contain amounts of biosynthetic intermediates, which are often found as fatty acyls in triacylglycerols (Bjostad and Roelofs 1984; Bjostad et al. 1987; Matsumoto et al. 2002). Indeed, the presence of these intermediates has facilitated the characterization of pheromone biosynthetic routes for many species of moth (Foster 2016; Jurenka 2020; Löfstedt et al. 2016). Although these intermediates can be abundant, for example (*Z*)-11-hexadecenoate (Z11-16:Acyl) in *Trichoplusia ni* (Bjostad and Roelofs 1983), in most species there is little evidence to suggest they are converted to pheromone in significant quantity. The major exception to this is the moth *Bombyx mori*, which stores a large quantity of the fatty acyl precursor in triacylglycerols, which is hydrolyzed and reduced to pheromone (bombykol) following the release of PBAN (Matsumoto 2010).

We have been studying pheromone biosynthesis in *Chloridea virescens* (Fabricius) (family: Noctuidae), with the aim of understanding how a finite amount of pheromone produced by a female moth may be released over time to maximize the chances of attracting a mate (Foster and Anderson 2020b; Foster et al. 2018). The sex pheromone of *C*. *virescens* consists (primarily) of a mixture of two aldehydes, (*Z*)-11-hexadecanal (Z11-16:Ald) and (*Z*)-9-tetradecenal (Roelofs et al. 1974). These are produced by typical Type 1 pheromone component biosynthesis, with the respective precursor alcohols, (*Z*)-11-hexadecenol (Z11-16:OH) and (*Z*)-9-tetradecenol, synthesized in gland cells before being oxidized to aldehydes in the cuticle (Choi et al. 2005; Teal and Tumlinson 1986). Using stable isotope tracer/tracee methods, we showed that Z11-16:Ald is turned over very rapidly [fractional synthetic rate (FSR), ca 100%/h] and is mostly produced rapidly from an intracellular pool of acetyl CoA (hereafter, referred to as the “direct route”), formed from mitochondrial β-oxidation of carbohydrate and fat (Foster 2009; Foster and Anderson 2015). However, a small amount (ca. 10%) of pheromone in our experiments lacked tracer and hence could not be produced from the same acetyl CoA pool. We assumed this portion was produced from previously synthesized precursor, possibly Z11-16:Acyl or 16:Acyl, stored in triacylglycerols (Foster et al. 2017) (hereafter, referred to as the “indirect route”), similar to that for production of bombykol in *B*. *mori* (Matsumoto 2010).

Since pheromone aldehydes are not synthesized wholly within gland cells, but are formed when precursor alcohols are translocated out of cells and oxidized in the cuticle (Fang et al. 1995; Teal and Tumlinson 1988), the rate of synthesis of Z11-16:Ald we determined must represent the rate of de novo biosynthesis of Z11-16:OH in the cell *plus* the rate of translocation out of the cell to the cuticular surface, during which Z11-16:OH is oxidized to Z11-16:Ald. Therefore, we wished to determine the rate of biosynthesis of intracellular Z11-16:OH, so as to determine whether the rate of translocation slows down production of Z11-16:Ald. Moreover, for the indirect route, stored fatty acyl precursors are likely hydrolyzed and converted to Z11-16:OH in order to produce Z11-16:Ald. Therefore, we expected that the dynamics of the intracellular Z11-16:OH pool should reflect both routes.

In this paper we explore the dynamics of Z11-16:Ald, its precursor alcohol, Z11-16:OH, and two stored fatty acyl moieties, Z11-16:Acyl and 16:Acyl. For this, we used stable isotope tracer/tracee techniques (Wolfe and Chinkes 2005) to track changes in the respective pools, while controlling pheromone biosynthesis (and amount of pheromone biosynthesized) through use of PBAN injections. Specifically, we addressed four questions:

i) How do the synthetic rates of Z11-16:OH and Z11-16:Ald compare?
ii) Is the direct/indirect route composition of the intracellular Z11-16:OH pool the same as that of Z11-16:Ald?
iii) Does stored Z11-16:Acyl contribute substantially to pheromone biosynthesis by the indirect route?
iv) Does the gland produce substantial quantities of stored 16:Acyl?

## Methods and Materials

### Insects

Our *C*. *virescens* colony was established originally from insects reared at USDA-ARS, Fargo, ND. Larvae were reared individually on a wheatgerm-casein diet (Shorey and Hale 1965). Pupae were sexed and the two sexes maintained in separate containers. Newly eclosed adult females were collected each day. For experiments with pupae, we estimated eclosion time by collecting insects less than 8 h after pupation and keeping them in timed cohorts.

### Gland Extraction and Chemical Analysis

Although the sex pheromone of *C*. *virescens* is a blend, we were interested in quantifying pheromone, not blend ratios. Therefore, we only quantified the major component, Z11-16:Ald, which is ca. 90% of pheromone mass (Roelofs et al. 1974), along with its precursor alcohol, Z11-16:OH, which may also be a minor pheromone component (Groot et al. 2018). Z11-16:Ald is found only in the cuticle, whereas Z11-16:OH is found almost entirely in gland cells (Foster and Anderson 2019; Groot et al. 2018). To extract these chemicals, the gland of an adult was extruded, excised, and placed in 25 μl of *n*-heptane along with 50 ng of (*E*)-11-tetradecenal (E11-14:Ald) as internal standard for at least 60 min at ambient temperature. For analysis of 16:Acyl and Z11-16:Acyl, we excised the gland as for pheromone extraction, but extracted it in 50 μl of dichloromethane: methanol (2:1) along with 200 ng of tripentadecanoin (internal standard) for at least 3 h at ambient temperature. For dissection of the gland in pupae, we first removed the terminal segments of the pupal cuticle before extruding and dissecting the gland. All analytes were identified by comparing their retention time and mass spectrum with authentic samples available in the laboratory.

Pheromone samples were analyzed directly from extract, whereas fatty acyl extracts were decanted away from the gland, the solvent evaporated, and the residue reacted with 0.5 M methanolic KOH for 15 min. After this, aqueous 1.0 M HCl was added, along with 50 μl of *n*-heptane, and the mixture briefly vortexed. The fatty acyl methyl esters (FAMEs) were in the top (*n*-heptane) layer.

Gas chromatograph/mass spectrometer (GC/MS) analyses were performed using an Agilent Technologies 7890B/5977A instrument. The GC used helium as carrier gas and was equipped with a splitless injector and a ZBWax capillary column (30 m × 0.25 mm i.d., 0.25 μm film thickness, Phenomenex, Torrance CA). For pheromone analyses, the flow rate was a constant 1.1 ml.min^−1^ and the column oven programmed from 80 °C (1 min delay) to 180 °C at 15 °C.min^−1^, then to 190 °C at 5 °C.min^−1^, and finally to 225 °C at 10 °C.min^−1^. For FAME analyses, the carrier gas was set to a constant 1.4 ml.min^−1^, and the column oven programmed from 80–180 at 20 °C.min^−1^, then to 190 °C at 2 °C.min^−1^, and finally to 225 °C at 20 °C.min^−1^.

### Insect Treatment and Mass Isotopomer Distribution Analysis (MIDA)

In most experiments, we introduced stable isotope label by allowing 1 d females to feed on a 12.5 μl drop of 10% U-^13^C-glucose solution (98% enrichment, Cambridge Isotope Laboratories, Cambridge, MA). However, in the experiment on fat accumulation, we injected 5 μl of 30% U-^13^C-glucose solution, into the abdomen of pupae or adults, using a calibrated glass capillary drawn to a fine point. For experiments testing the effect of PBAN, females were injected (again using a glass capillary) with 5 pmol of PBAN (synthetic peptide with an amino acid sequence of that of *Helicoverpa zea*, Bachem, Torrance, CA) in saline (1 μl; 187.5 mM NaCl, 4.83 mM KCl, 2.6 mM CaCl_2_, 10 mM Hepes, 14 mM glucose, adjusted to pH 6.8) or saline only (control).

MIDA is a binomial solution for determining enrichment of an isotopically labeled monomer in a polymeric product (Hellerstein and Neese 1992; Wolfe and Chinkes 2005). Essentially, labeled monomer (in our case, ^13^C_2_-acetate from U-^13^C-glucose) is introduced and GC/MS used to determine the intensities of several isotopomers of the polymeric products, Z11-16:Ald, Z11-16:OH, Z11-16:Acyl, and 16:Acyl, all octomers of acetyl CoA: without a labeled monomer (M + 0), and with one (M + 1) or two (M + 2) labeled monomers. Then, tracer to tracee (TTR) ratios are calculated, allowing for the natural abundances of stable isotopes and the overlap of isotopomer spectra.

- (i) $$TTR\left(M+1\right)={\left(M+1/M+0\right)}_{post}-{\left(M+1/M+0\right)}_{pre}$$
- (ii) $$TTR\left(M+2\right)={\left(M+2/M+0\right)}_{post}-{\left(M+2/M+0\right)}_{pre} -d{T}_{1}\times TTR\left(M+1\right)$$

The ‘pre’ and ‘post’ terms are isotopomer intensities before and after label enters the system, while dT_1_ accounts for the spectral overlap of the (M + 1) and (M + 2) isotopomers.

Precursor enrichment (*PE*; the proportion of labeled monomer in all isotopomers of the product) is then calculated, with n (= 8 in our calculations) the number of monomers in the polymer:

- (iii) $$PE=2\times \left[TTR\left(M+2\right)/TTR\left(M+1\right)\right]\div \left[\left(n-1\right)+TTR\left(M+2\right)/TTR\left(M+1\right)\right]$$

Fractional synthesis rate (FSR) of a polymer is generally calculated as the rate of change of an enriched product over time divided by its final (equilibrium) enrichment. If we determine this using the change of enrichment of singly labeled polymer (E_1_), usually the most abundant of the isotopomers with label, we need to use the enrichment of this product (E_p1_). However, *PE* is enrichment of the entire suite of isotopomers and must be converted to E_p1_ by a binomial expansion [equation (iv)]. Then, FSR and absolute synthetic rate (ASR) can be calculated by equations (v) and (vi), respectively (Wolfe and Chinkes 2005).

- (iv) $${E}_{p1}=n\times PE\times {\left(1-PE\right)}^{n-1}$$
- (v) $$FSR=\left[{E}_{1\left(t2\right)}-{E}_{1\left(t1\right)}\right]/\left({t}_{2}-{t}_{1}\right)\times {E}_{p1}$$
- (vi) $$ASR=FSR\times product pool size$$

With the mass spectrometer in selected ion mode, we recorded the intensities of (M + 0), (M + 1) and (M + 2) isotopomers of Z11-16:Ald, Z11-16:OH, methyl Z11-16:Acyl and methyl 16:Acyl from ions with intact carbon skeletons, along with *m/z* 192 (M^+^-18; loss of H_2_O from E11-14:Ald) and *m/z* 256 (M^+^ for methyl pentadecanoate) of the internal standards for quantitation. These were *m/z* 220, 222 and 224 (M^+^-18; loss of H_2_O from Z11-16:Ald), 222, 224, and 226 (M^+^-18; loss of H_2_O from Z11-16:OH), *m/z* 268, 270 and 272 (M^+^ for methyl Z11-16:Acyl), and *m/z* 270, 272 and 274 (M^+^ for methyl 16:Acyl). For MIDA calculations, we set an integration limit of 50 units for isotopomers of Z11-16:Ald, Z11-16:OH and Z11-16:Acyl and a limit of 200 units for 16:Acyl. This was done, because small ion intensities [especially for (M + 2) isotopomers] can introduce large errors in the *PE* calculations (see equation iii).

As well as calculating FSR and ASR for Z11-16:OH and Z11-16:Ald, we used MIDA to calculate the amount of polymer biosynthesized de novo after label was introduced (hereafter denoted or subscripted by “DN”), and the amount of polymer existing at the start of an experiment or synthesized during an experiment from a complete 16-carbon precursor prior to label being introduced (i.e., lacking label and hereafter denoted or subscripted by “EX”). For our analytes, the former represents de novo synthesis from the mixed acetyl CoA tracer/tracee pool (i.e., direct route), while the latter represents analyte synthesized prior to label being introduced plus any synthesis by the indirect route. In some experiments, we fed females and immediately decapitated them, and later injected them with PBAN. This meant there were negligible amounts of Z11-16:Ald (both DN and EX) at the start of the experiment, making synthesis of Z11-16:Ald_EX_ from the indirect route more apparent. For these calculations, *PE* allowed prediction of the relative intensities of all nine isotopomers of an analyte. We then used the recorded M + 2 isotopomer intensity (minus spectral contributions from the M + 0 and M + 1 isotopomers) to calculate the expected intensities of the nine isotopomers. These were summed and used to quantify the amount of analyte_DN_. The intensity of the M + 0 isotopomer from this calculation was then subtracted from the recorded intensity of the M + 0 isotopomer and the difference used to quantify the amount of analyte_EX_.

### Experiments

#### Synthetic rates of Z11-16:Ald and Z11-16:OH

Two hours into the scotophase, 1 d females were each fed a drop of U-^13^C-glucose and left for 0, 2, 4, 7.5, 11, 15, 22.5, 30, 45, 60 or 90 min before glands were extracted and analyzed by MIDA for Z11-16:Ald and Z11-16:OH. Six to eight females were analyzed for each time point. For determination of pool sizes (to calculate ASR), we extracted glands of unfed 1 d-old females (n = 6) 2 h into the scotophase.

#### Dynamics of Z11-16:OH and Z11-16:Ald in intact females

We recorded the dynamics of DN and EX Z11-16:OH and Z11-16:Ald using MIDA in 1 d females fed U-^13^C-glucose, starting 2 h into the scotophase. Two types of females were used: intact (Intact) and intact injected with 5 pmol PBAN (Intact + PBAN) immediately after feeding. Intact + PBAN females were used because we wanted to see whether the increased amount of pheromone produced (Foster and Anderson 2020a) corresponded with increased amount of Z11-16:OH. Females (n = 10–11 for each time point) were analyzed 15, 30 and 60 min after feeding.

#### Dynamics of Z11-16:Ald and Z11-16:OH in decapitated females

Next, we tested the dynamics of DN and EX Z11-16:OH and Z11-16:Ald using 1 d females fed U-^13^C-glucose, immediately decapitated after feeding and left for 16 h. Females (n = 7–11 for each time point) were then injected with 5 pmol PBAN and sampled at various times up to 180 min following PBAN injection.

#### Dynamics of acyl intermediates

For dynamics of Z11-16:Acyl and 16:Acyl (both DN and EX), we used Intact and Intact + PBAN females, as for the third experiment. Both types (n = 10–15 for each time point) were sampled 30, 60, 120 and 180 min after feeding. Intact females were also sampled at 0 min and 960 min after feeding. Intact + PBAN females were not sampled at 960 min because the effect of a bolus of PBAN on pheromone biosynthesis only lasts for a few hours (see Results).

#### 16:Acyl and Z11-16:Acyl accumulation in the gland

First, we determined the quantities of Z11-16:Acyl and 16:Acyl in the gland of different age female pupae and adults: pupae 2 d prior to eclosion (2d-P), pupae 1 d prior to eclosion (1d-P), adults that had eclosed within 3 h (A + 0d), and adults that had eclosed 1 d (A + 1d) and 2 d (A + 2d) prior. In this (and subsequent experiments), we dissected the pheromone gland more carefully than before, ensuring that we collected as little extraneous tissue as possible. Fats in the gland were extracted, converted to FAMEs, and quantified by GC/MS.

Next, pupae (2d-P) or adults (A-0d) were injected with 5 μl U-^13^C-glucose and left for the requisite amount of time until 1 d adults. Fats were extracted, converted to FAME and Z11-16:Acyl and 16:Acyl analyzed by GC/MS for calculation of *PE* by MIDA. Finally, 2d-P, pupae that would eclose within 6 h (0d-P), and A + 0d were injected with 5 μl U-^13^C-glucose and left for the requisite time until 1 d adults. Then, the pheromone gland was dissected, extracted and analyzed by GC/MS for calculation of *PE* of Z11-16:Ald.

### Statistical analyses

Except for the synthetic rate experiment (i) and the fat accumulation experiments (v), data were analyzed by analysis of covariance in JMP Pro (JMP 2020), using insect treatment as the categorical variable, after first confirming that data were normally distributed and that there was homogeneity of variance (see Results for the variables and effects tested). For the synthetic rate experiment (i), *PEs* of Z11-16:Ald and Z11-16:OH at each time point were compared by ANOVA. In the third experiment, amounts of each of the moieties were compared across time by Tukey–Kramer HSD tests, with α = 0.05. In the fat accumulation experiment, data were analyzed by either ANOVA or non-parametric tests, depending on whether data were normally distributed or not.

## Results

### Synthetic rates of Z11-16:Ald and Z11-16:OH

**Fig. 1:**
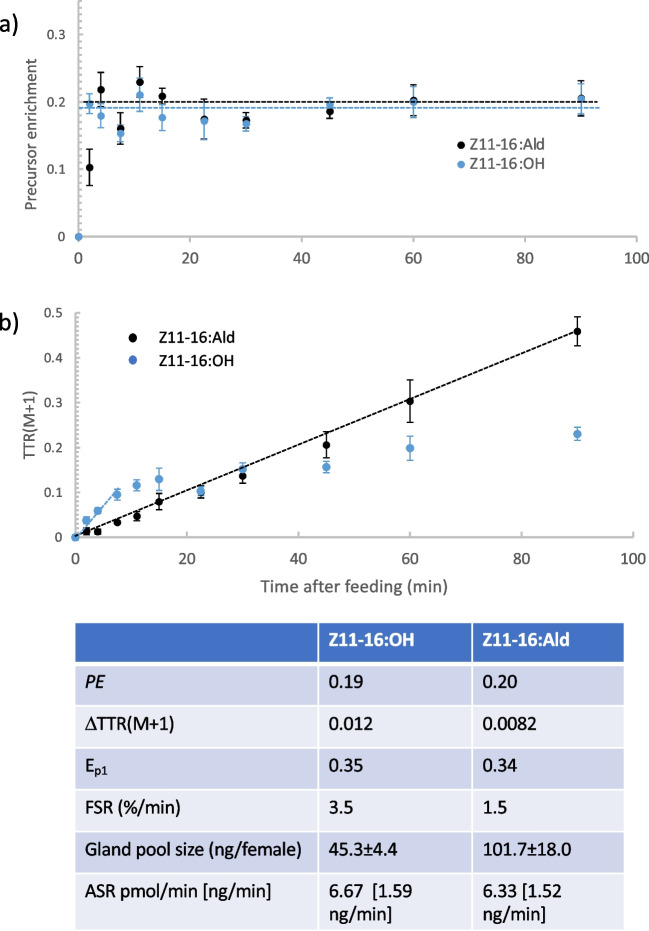
Mass isotopomer distribution analysis of precursor enrichment (*PE*), fractional (FSR) and absolute (ASR) synthetic rates of (*Z*)-11-hexadecenal (Z11-16:Ald) and (*Z*)-11-hexadecenol (Z11-16:OH) in the pheromone gland of female *Chloridea virescens*. (**a**) Change of *PE* after moths had fed on U-^13^C-glucose and (**b**) change of intensity of isotopomers with one ^13^C_2_-acetate monomeric unit after correction for natural isotopic abundances [TTR(M + 1)]. In (**a**) the dashed lines are asymptotes of the equilibrated *PE*s, while in (**b**) the dashed lines are initial ∆TTR(M + 1) used in the calculations. The table summarizes the parameters calculated. E_P1_ is the enrichment of isotopomers with one^13^C_2_-acetate monomeric unit used with ∆TTR(M + 1) to calculate FSR (see text)

*PE* for both Z11-16:OH_DN_ and Z11-16:Ald_DN_ came to rapid equilibration within 2.5–10 min (Fig. 1a). Initially, *PE* of Z11-16:OH was greater (2.5 min after feeding, ANOVA, F_1,10_ = 9.60, p = 0.01) than that of Z11-16:Ald, consistent with the alcohol being synthesized before the aldehyde; however, at no other time were the respective *PE*s different. For both products, the initial increases in *PE* were very rapid before plateauing at < 10 min. Equilibrated *PE*s were estimated from asymptotes of 3-parameter exponential models (JMP 2020) (Fig. 1a). Calculation of the rate of change of singly labeled product [TTR(M + 1)] was straightforward for Z11-16:Ald, as the response was linear throughout (Fig. 1b). However, calculation for Z11-16:OH was more problematic since the linear increase was brief (< 11 min), before plateauing. Consequently, we used a linear fit over the first 7.5 min after feeding to obtain the rate (Fig. 1b). This is a problem for MIDA encountered when measuring fast turnover rates (Chinkes et al. 1996).

After calculation of the respective enrichments of singly labeled products (equation iv), FSRs were calculated (equation v) to be 3.5%.min^−1^ (211%. h^−1^) and 1.5%.min^−1^ (90%.h^−1^) for Z11-16:OH and Z11-16:Ald, respectively (Fig. 1). Using their respective pool sizes (Fig. 1), very similar ASR values (equation vi) of 1.59 ng.min^−1^ (6.67 pmol.min^−1^) and 1.52 ng.min^−1^ (6.33 pmol.min^−1^) were calculated for Z11-16:OH and Z11-16:Ald, respectively (Fig. 1).

### Dynamics of Z11-16:OH and Z11-16:Ald in intact females

**Fig. 2:**
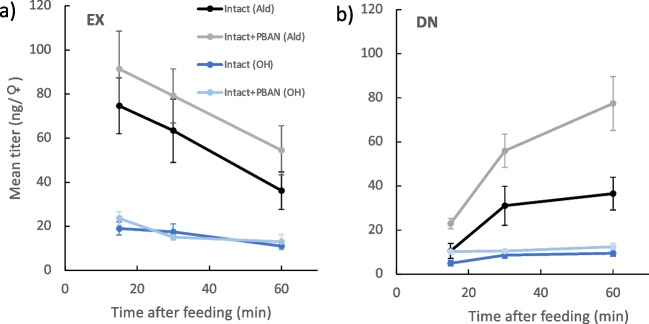
Changes in titers of (*Z*)-11-hexadecenal (Ald) and (*Z*)-11-hexadecenol (OH) in 1 d *Chloridea virescens* females following feeding on U-^13^C-glucose. Two types of females were used: normal intact (Intact) and females injected with 5 pmol of pheromone biosynthesis-activating neuropeptide (Intact + PBAN) immediately after feeding. (**a**) Mean amount of compound remaining that had been synthesized prior to the start of the experiment plus the amount synthesized during the experiment from a complete 16-carbon metabolite synthesized prior to label being introduced (EX), and (**b**) mean amount of compound synthesized de novo from the acetate pool after label was introduced (DN). SEMs are given

Z11-16:Ald_EX_ titer in Intact and Intact + PBAN females declined over the experiment (Fig. 2a). ANCOVA showed an effect of time (F = 9.46, p = 0.003), but not of female type (F = 2.72, p = 0.10) and no interaction between terms (F = 0.006, p = 0.94). By contrast, Z11-16:Ald_DN_ increased with time (F = 24.6, p < 0.001) and differed between female type (F = 17.4, p < 0.001), with Intact + PBAN females having more Z11-16:Ald_DN_ than Intact females (Fig. 2b). There was no interaction between terms (F = 3.36, p = 0.07). Thus, for both types of female, Z11-16:Ald_EX_ was rapidly replaced by Z11-16:Ald_DN_, with Intact + PBAN females making more Z11-16:Ald_DN_ than Intact females.

Similar to Z11-16:Ald, although less striking, Z11-16:OH_EX_ declined (F = 10.9, p = 0.002) over time, but no effect of female type (F = 0.41, p = 0.53) and no interaction between terms (F = 0.06. p = 0.81) was observed (Fig. 2a). Z11-16:OH_DN_ increased with time (ANCOVA, F = 6.75, p = 0.01) and showed a difference between female type (ANCOVA, F = 11.1, p = 0.001), with Intact + PBAN females having greater amounts than Intact females (Fig. 2b). There was no interaction (F = 0.58, p = 0.45) between terms. In comparison to Z11-16:Ald, EX was replaced more slowly by DN.

### Dynamics of Z11-16:Ald and Z11-16:OH in decapitated females

**Fig. 3:**
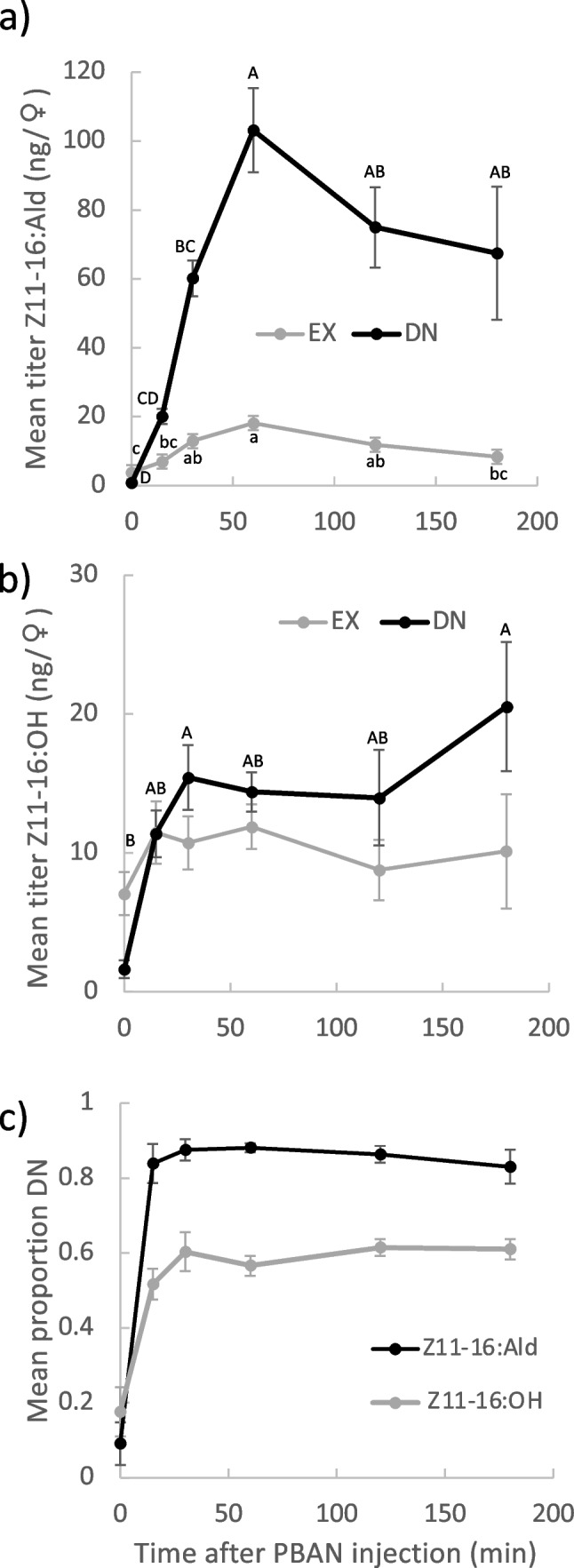
Changes of pheromone gland metabolites in female *Chloridea virescens* females that were decapitated immediately after feeding on U-^13^C-glucose and then left for 16 h before being injected with 5 pmol of pheromone biosynthesis-activating neuropeptide. Mean titers of (**a**) of (*Z*)-11-hexadecenal (Z11-16:Ald) and (**b**) of (*Z*)-11-hexadecenol (Z11-16:OH). DN is the amount of compound synthesized de novo from the acetate pool after label was introduced, while EX is the amount of compound remaining that had been synthesized prior to the start of the experiment plus the amount synthesized during the experiment from a complete 16-carbon metabolite synthesized prior to label being introduced. (**c**) Ratios of DN (out of total amount of DN + EX) over time. SEMs are given. In (**a**) and (**b**), different letters of the same case indicate means of a moiety that are different among times (p < 0.05; Tukey–Kramer HSD test); in (**b**) there was no difference among times for Z11-16:OH_EX_ titer

Females fed U-^13^C-glucose and decapitated 16 h before being injected with PBAN, showed a rapid increase (F_5,52_ = 14.17, p < 0.001) in amount of Z11-16:Ald_DN_, from almost none to a peak amount of ca 100 ng, 60 min after injection (Fig. 3a). These females had relatively small amounts of Z11-16Ald_EX_ throughout the experiment (Fig. 3a), although amount increased slightly (F_5,52_ = 7.16, p < 0.001), with a peak at 60 min, before declining by 180 min (Tukey–Kramer HSD test). Z11-16:OH_DN_ amount also increased (F_5,52_ = 3.64, p = 0.007) from 0–30 min (Tukey–Kramer HSD test), before plateauing (Fig. 3b). By contrast, Z11-16:OH_EX_ amount was constant (F_5,52_ = 0.65, p = 0.66) over the experiment (Fig. 3b).

Proportions (out of total) of Z11-16:Ald_DN_ and Z11-16:OH_DN_ (Fig. 3c) both initially increased then plateaued 30 min after PBAN injection; however, the plateau for the alcohol (ca. 0.6) was lower than that for the aldehyde (ca. 0.85–0.9; Fig. 3). That is, less EX was replaced by DN for Z11-16:OH than for Z11-16:Ald.

### Dynamics of acyl intermediates

**Fig. 4:**
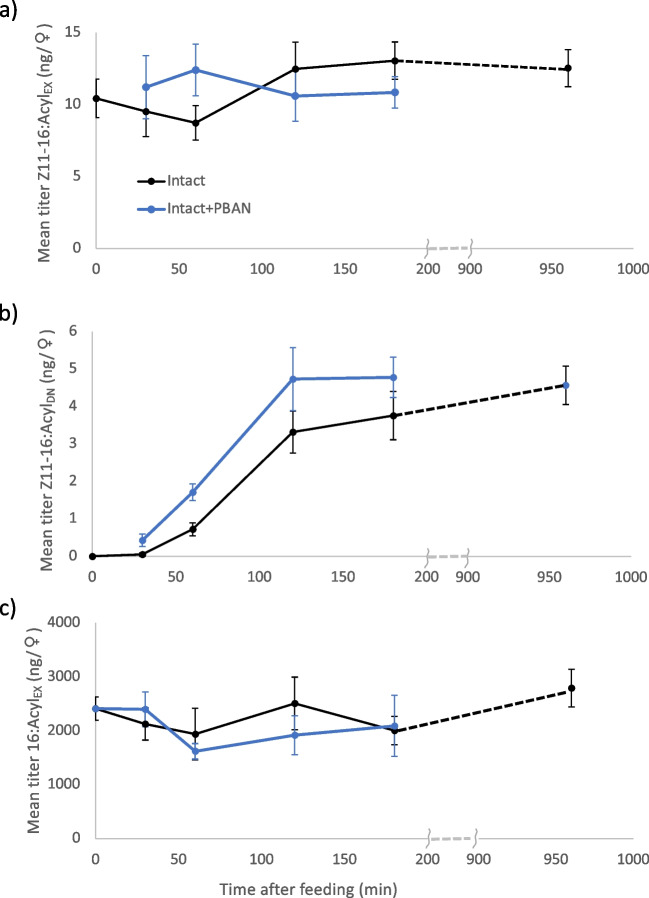
Changes in fatty acyl moieties in the pheromone gland of *Chloridea virescens* females following feeding on U-^13^C-glucose. Two types of females were used: intact (Intact) and intact females injected with 5 pmol of pheromone biosynthesis-activating neuropeptide (Intact + PBAN) immediately after feeding. (**a**) Mean titers of (*Z*)-11-hexadecenoate synthesized prior to start of the experiment (before label introduced) plus the amount synthesized during the experiment from a complete 16-carbon metabolite synthesized prior to label being introduced (Z11-16:Acyl_EX_), (**b**) mean titers of (*Z*)-11-hexadecenoate synthesized de novo from the acetate pool after label was introduced (Z11-16:Acyl_DN_) and (**c**) mean titers of hexadecanoate synthesized prior to the start of the experiment (before label introduced; 16:Acyl_EX_). There was no consistent detectable level of de novo-synthesized hexadecanoate. SEM are given

Amount of Z11-16:Acyl_EX_ did not change during the experiment for both Intact and Intact + PBAN females (Fig. 4a). When sample points unique to Intact females (i.e., at t = 0 and 1440 min) were excluded, ANCOVA showed no effect of time (F = 1.09, p = 0.30) or insect type (F = 0.07, p = 0.79), or any interaction between terms (F = 3.05, p = 0.09). By contrast, amount of Z11-16:Acyl_DN_ showed effects of both time (F = 82.7, p < 0.001) and insect type (F = 6.64, p = 0.01), with no interaction (F = 0.32, p = 0.57) between terms. Essentially, amount of Z11-16:Acyl_DN_ increased over time for both types of females, but increased more for Intact + PBAN ones (Fig. 4b). No change in Z11-16:Acyl_EX_ (ANOVA, F_1,39_ = 0.34, p = 0.56) and an increase in Z11-16:Acyl_DN_ (F_1,39_ = 68.7, p < 0.001) were apparent at 960 min for Intact females (Fig. 4a,b).

We found little evidence for consistent detection of 16:Acyl_DN_ in the experiment. Only 9/98 samples (across both types of females, and at various times) were calculated to have miniscule amounts of this moiety (data not shown). Therefore, virtually all, if not all, stored 16:Acyl in the gland was synthesized before ^13^C tracer was introduced. The amounts of 16:Acyl_EX_ were much greater (ca. 200 ×) than the amounts of Z11-16:Acyl_EX_ (Fig. 4a,c). ANCOVA showed no effect of time (F = 2.7, p = 0.11) or type of female (F = 0.70, p = 0.41) and no interaction between terms F = 0.15, p = 0.7). That is, the amount of 16:Acyl_EX_ showed no change throughout the experiment, regardless of female type. Even by 960 min, 16:Acyl_EX_ amount had not changed in Intact females (ANOVA, F_1,41_ = 0.02, p = 0.88).

### 16:Acyl and Z11-16:Acyl accumulation in the gland

**Fig. 5:**
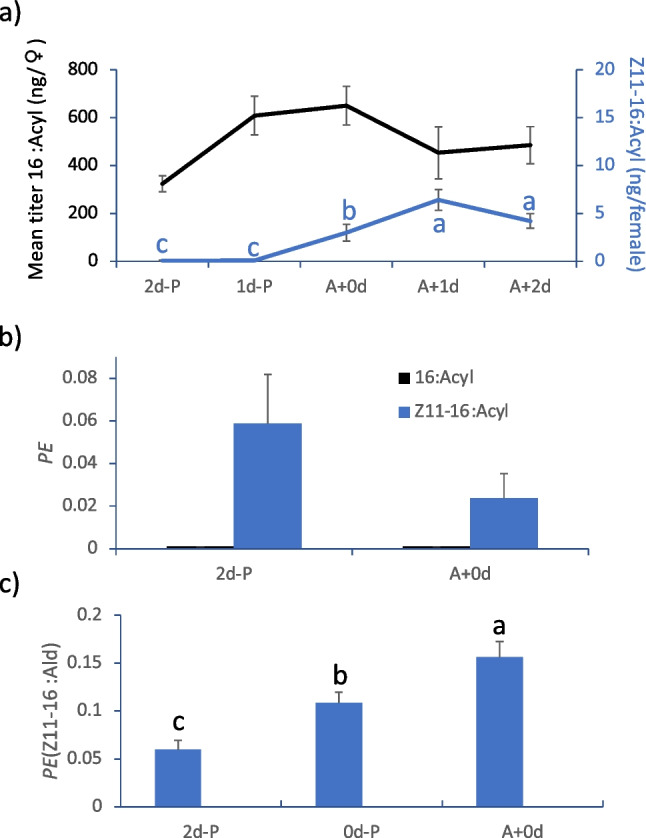
Titers and enrichments of fatty acyl moieties and pheromone in pupae and adult female *Chloridea virescens*. (**a**) Mean titers of (*Z*)-11-hexadecenoate (Z11-16:Acyl) and hexadecanoate (16:Acyl). Females were analyzed as pupae 2 d prior to eclosion (2d-P), pupae 1 d prior to eclosion (1d-P), adults that eclosed within 3 h (A + 0d), and adults that eclosed 1 d (A + 1d) and 2 + d (A 2d) prior. Precursor enrichments (*PE*) of (**b**) 16:Acyl and Z11-16:Acyl and (**c**) (*Z*)-11-hexadecenal (Z11-16:Ald) of different age *C*. *virescens* females injected with U-.^13^C-glucose and analyzed when 1 d adults. 2d-P = injected 2 d before eclosion, 0d-P = injected within 6 h of eclosion, A + 0d = injected within 6 h after eclosion. SEM are given. Different letters indicate means that are different (p < 0.05) in (**a**) by paired Wilcoxon tests, and in (**c**) by Student’s t-test. There were no differences among treatments in (**b**)

There were no differences in amount of methyl 16:Acyl in base-methanolized glands among any of the different-aged females (ANOVA, F_4,40_ = 2.26, p = 0.079). By contrast, methyl Z11-16:Acylmethyl amount increased with age, with none detected in pupae, an intermediate amount in newly eclosed adults, and the greatest amounts in 1 d and 2 d adults (paired Wilcoxon tests; Fig. 5a).

When U-^13^C-glucose was injected into pupae (2d-P) or adults (A + 0d), no enrichment was detected in 16:Acyl of 1 d adults. By contrast, we detected enrichment of methyl Z11-16:Acyl in 1 d adults for both types of females (enrichment not different whether injected into adults or pupae, ANOVA, F_1,15_ = 2.21, p = 0.16) (Fig. 5b).

Finally, when U-^13^C-glucose was injected into pupae (2d-P, 0d-P) or newly eclosed adults (A + 0d) and analyzed when 1 d adults, Z11-16:Ald enrichment was detected and increased in the order A + 0d > 0d-P > 2d-P (ANOVA, F_2,26_ = 13.9, p < 0.001) (Fig. 5c).

## Discussion

### How do the synthetic rates of Z11-16:OH and Z11-16:Ald compare?

The FSR/ASR experiment demonstrated that intracellular Z11-16:OH and cuticular Z11-16:Ald pools had similar ASRs. Given that the ASR of Z11-16:Ald, in effect, represents the rate of synthesis of Z11-16:OH plus its rate of translocation and oxidation, we can conclude that translocation and oxidation do not significantly slow production of Z11-16:Ald once Z11-16:OH has been biosynthesized de novo. Although the ASRs of the two moieties are similar, the FSRs are quite different, with the smaller Z11-16:OH pool being turned over more rapidly than the larger Z11-16:OH pool. In fact, given that the de novo-produced Z11-16:OH pool represents only ca. 60% of the total intracellular Z11-16:OH (see next section), then its FSR must be some 170% faster than that calculated. The rate of change of TTR(M + 1) for Z11-16:OH was decreasing 7.5 min after feeding (the last point used to construct the line), which could have resulted in our underestimating FSR.

### Is the direct/indirect route composition of the intracellular Z11-16:OH pool the same as that of Z11-16:Ald?

**Fig. 6:**
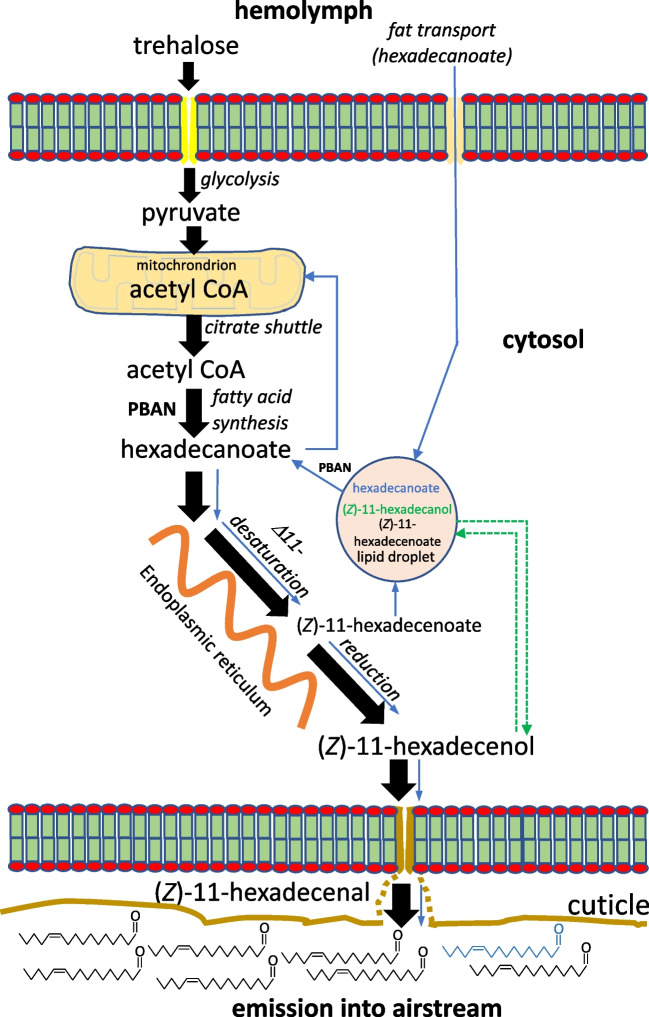
Scheme of the dual pool synthesis of (*Z*)-11-hexadecenal in *Chloridea virescens*. The major, direct route involving de novo synthesis of hexadecanoate is indicated by thick black arrows while the minor, indirect route, involving recirculation of stored hexadecanoate, is indicated by thin blue arrows. The two pools of (*Z*)-11-hexadecenol are indicated, with the less labile pool suggested to occur in lipid droplets along with other neutral lipids. See text for more discussion

Results presented here and elsewhere (Foster et al. 2017) show that most Z11-16:Ald is formed by a direct route of de novo biosynthesis from acetyl CoA, with a small portion (10–15%) produced via an indirect route, in which an intermediate with a 16-carbon skeleton is stored and converted later to Z11-16:Ald (Fig. 6). We assumed that the intracellular pool of precursor Z11-16:OH should also exhibit this. However, while stimulation of decapitated females by PBAN gave a rapid increase in both Z11-16:OH_DN_ and Z11-16:Ald_DN_, the former plateaued at a substantially lower proportion (ca. 0.6) of the total pool than did the latter (ca. 0.85). This demonstrates that intracellular Z11-16:OH is not spatially homogeneous in the cell, with Z11-16:OH_DN_ being translocated preferentially over Z11-16:OH_EX_. One explanation for this is that, once de novo biosynthesis is started, Z11-16:OH_DN_ is shuttled rapidly from the endoplasmic reticulum to the cell membrane in a flux that effectively favors its translocation over that of Z11-16:OH_EX_ from the cell. Alternatively, Z11-16:OH_EX_ may be compartmentalized and not available for translocation. The latter is supported by the decapitation experiment, in which a substantial amount of Z11-16:OH_EX_ remained (c.f., Z11-16:Ald_EX_) 18 h after decapitation.

When PBAN was injected into decapitated females, there was a very rapid increase in Z11-16:Ald_DN_, accompanied by an increase in Z11-16:OH_DN_, confirming the effect of PBAN on de novo biosynthesis (Eltahlawy et al. 2007). However, we also observed a slight increase in the amount of Z11-16:Ald_EX_, with no change in Z11-16:OH_EX_. This is consistent with the indirect route being influenced by PBAN, with the lack of change in Z11-16:OH_EX_ amount likely due to its replacement by hydrolysis of stored precursor fat. Thus PBAN, in addition to its primary role of influencing de novo pheromone synthesis, seems to cause some lipolysis and reduction of fats, similar to the situation in *B*. *mori* (Matsumoto 2010).

### Does stored Z11-16:Acyl contribute substantially to pheromone biosynthesis by the indirect route?

Stored Z11-16:Acyl is the most obvious candidate for the indirect route production of Z11-16:Ald. In our experiment with Intact and Intact + PBAN females, we observed an increase in Z11-16:Acyl_DN_, with Intact + PBAN females having more than Intact ones. Combined with a similar difference in Z11-16:Ald_DN_, this is consistent with a greater rate of pheromone biosynthesis occurring in the former females. For Intact females, Z11-16:Acyl_DN_ amount increased over the whole 960 min of the experiment, while the amount of Z11-16:Acyl_EX_ did not change. Together, these suggest that once Z11-16:Acyl is stored in glycerolipids, it contributes little, if at all, to biosynthesis of Z11-16:Ald_EX_ by the indirect route. This is not to say that Z11-16:Acyl, nor indeed all stored acids, are irreversibly stored in glycerolipids. Rather, it indicates that the rate of lipolysis in the gland is low, with more abundant acids more likely to be lipolyzed. Given its much greater abundance, it seems more likely that stored 16:Acyl is hydrolyzed, desaturated, reduced and oxidized to Z11-16:Ald through the indirect route (Fig. 6). However, due to the large amount of this acid in the gland, along with its likely presence in extraneous tissue in our dissections, we were unable to detect the small changes in titer that would support this contention.

### Does the gland produce substantial quantities of stored 16:Acyl?

With its ability to synthesize fatty acyl derivatives de novo, one might assume that the abundance of 16:Acyl (and other common fatty acids) in the gland of *C*. *virescens* (Foster 2005) and many other moth species (Bjostad et al. 1987) is a consequence of the gland’s synthetic ability. However, our experiments show that the gland does not synthesize significant amounts of stored 16:Acyl at least 2 days prior to, and after, commencing pheromone biosynthesis. This contrasted with the production of both labeled Z11-16:Ald and stored Z11-16:Acyl when pupae injected with U-^13^C-glucose were analyzed as adults. Further, the detection of relatively high amounts of 16:Acyl prior to synthesis of pheromone suggests that this acid (and possibly other common fatty acids) is synthesized before the gland is functional, and is likely transported to the gland during development. Although limited lipolysis (see above) in the gland may liberate a small amount of 16:Acyl for pheromone biosynthesis via the indirect route, it seems that the major role of stored fats in the gland is mitochondrial β-oxidation to generate acetyl CoA for synthesis of pheromone (Foster and Anderson 2015) or ATP production.

## Data Availability

Data available on request to the lead author.
